# Live Imaging of the Zebrafish Embryonic Brain by Confocal Microscopy

**DOI:** 10.3791/1217

**Published:** 2009-04-01

**Authors:** Ellie Graeden, Hazel Sive

**Affiliations:** Department of Biology, MIT - Massachusetts Institute of Technology; Whitehead Institute for Biomedical Research, MIT - Massachusetts Institute of Technology

## Abstract

In this video, we demonstrate the method our lab has developed to analyze the cell shape changes and rearrangements required to bend and fold the developing zebrafish brain (Gutzman et al, 2008). Such analysis affords a new understanding of the underlying cell biology required for development of the 3D structure of the vertebrate brain, and significantly increases our ability to study neural tube morphogenesis. The embryonic zebrafish brain is shaped beginning at 18 hours post fertilization (hpf) as the ventricles within the neuroepithelium inflate. By 24 hpf, the initial steps of neural tube morphogenesis are complete. Using the method described here, embryos at the one cell stage are injected with mRNA encoding membrane-targeted green fluorescent protein (memGFP). After injection and incubation, the embryo, now between 18 and 24 hpf, is mounted, inverted, in agarose and imaged by confocal microscopy. Notably, the zebrafish embryo is transparent making it an ideal system for fluorescent imaging. While our analyses have focused on the midbrain-hindbrain boundary and the hindbrain, this method could be extended for analysis of any region in the zebrafish to a depth of 80-100 μm.

**Figure:**
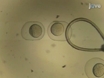


## Protocol

### 1. Preparing the mRNA for Injection

1. The mRNA used in this procedure is transcribed from a plasmid encoding CAAX-eGFP (memGFP) mRNA. First linearize the plasmid according to Gutzman et al, 2008.
2. Then transcribe memGFP mRNA using the mMessage mMachine kit.
3. Dilute the resulting mRNA to 1μg/μl, aliquot, and store at -80°C.
4. Prepare an injection mold of 1% agarose with lanes the width of embryos at the one cell stage.
5. On the day before injection, set up mating cages separating the male from the females.
6. On the day of injection, pull capillary needles using a micropipette puller to prepare for injection.

### 2. Injecting the mRNA

1. On the day of injection, thaw an aliquot of 1μg/μl memGFP mRNA on ice.
2. Dilute the mRNA 1:5 in water to a final concentration of 200ng/μl, and keep on ice.
3. Load the capillary needle with 1μl of prepared memGFP mRNA at 200ng/μl.
4. Place loaded needle into a micromanipulator attached to a gas-powered microinjector.
5. Adjust the injection volume to 1nl.
6. Pull the dividers on wild type fish set up in mating cages from the previous day.
7. Collect the embryos as soon as they are laid. Orient them in the agarose injection mold covered by embryo medium (Westerfield, 1995) with the single cell on the side away from the micromanipulator.
8. Inject through the chorion and yolk such that the mRNA is deposited directly into the single cell.  Inject approximately 50 embryos per experiment. Do not inject if the cell has divided.
9. Incubate embryos at 28°C overnight in embryo medium

### 3. Mounting and Imaging Embryos

1. To mount and image the embryos, remove the chorion with forceps from the embryos under a stereomicroscope one hour before the time point of interest.
2. Prepare a slide for mounting up to 4 embryos.
  - Use silicone vacuum grease to seal a coverslip on the underside of a specially designed 2mm-thick plastic slide with a hole approximately 1cm in diameter.
  - Fill the hole in the slide with 0.7% agarose, and let stand about 20 minutes or until hardened
  - Once the agarose has solidified, remove a small plug using 200μl pipette tips to form cylindrical holes in which to mount each embryo.
3. Place the embryos on the agarose-filled slide under a dissecting microscope. Add 50μl of tricaine (Westerfield, 1995) to anesthetize the embryos.
4. Use forceps to position the embryos in the cylindrical holes in the agarose with the brain or region of interest against the underlying coverslip.
5. Cover the embryos in the agarose with another coverslip secured with silicone vacuum grease.
6. Image the embryo using an inverted, fluorescent, laser-scanning or spinning disk confocal microscope. Image at 63x or higher to collect high resolution images of single cells within the neuroepithelium.
7. Images are exported as TIFF files from the LSM software and analyzed using Photoshop.

### 4. Representative Results/Outcome

Shown in Figure 1 is a representative confocal image of a 24 hpf zebrafish neuroepithelium between the midbrain and hindbrain ventricles. Each cell is labeled with membrane-bound GFP.


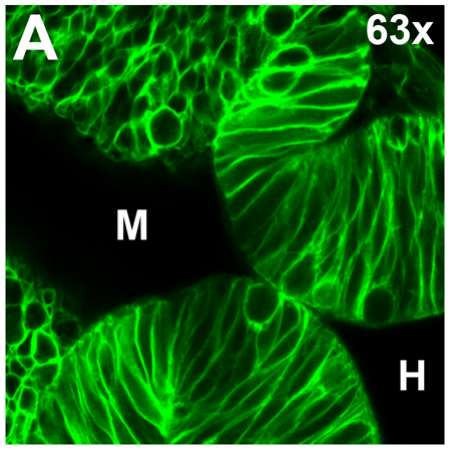
 **Figure 1. Live confocal imaging of a memGFP-injected 24 hpf zebrafish embryo.** (A) Neuroepithelium of a 24 hpf embryo with each cell outlined by GFP. Region imaged separates the midbrain and hindbrain ventricles (M, H respectively), forming the midbrain-hindbrain boundary.

## Discussion

In this video, we demonstrate a method for mRNA injection into single cell zebrafish embryos. Here, we use mRNA encoding a membrane-targeted GFP to label each cell. We then demonstrate how to mount and image the developing brain at single cell resolution. This technique has allowed us to study a novel type of cell shape change, basal constriction, required for formation of the midbrain-hindbrain boundary (Gutzman et al, 2008). Similar analysis of other phenomena has the potential to significantly expand our understanding of vertebrate morphogenesis and the underlying cell biology.
